# Highlights on the capacities of "Gel-based" proteomics

**DOI:** 10.1186/1477-5956-8-23

**Published:** 2010-04-28

**Authors:** François Chevalier

**Affiliations:** 1 Proteomic Laboratory, iRCM, CEA, Fontenay aux Roses, France

## Abstract

Gel-based proteomic is the most popular and versatile method of global protein separation and quantification. This is a mature approach to screen the protein expression at the large scale, and a cheaper approach as compared with gel-free proteomics. Based on two independent biochemical characteristics of proteins, two-dimensional electrophoresis combines isoelectric focusing, which separates proteins according to their isoelectric point, and SDS-PAGE, which separates them further according to their molecular mass. The next typical steps of the flow of gel-based proteomics are spots visualization and evaluation, expression analysis and finally protein identification by mass spectrometry. For the study of differentially expressed proteins, two-dimensional electrophoresis allows simultaneously to detect, quantify and compare up to thousand protein spots isoforms, including post-translational modifications, in the same gel and in a wide range of biological systems. In this review article, the limits, benefits, and perspectives of gel-based proteomic approaches are discussed using concrete examples.

## Introduction

Proteomics, one of the most important areas of research in the post-genomic era, is not new in terms of its experimental foundations [1]. It is a natural consequence of the huge advances in genome sequencing, bioinformatics and the development of robust, sensitive, reliable and reproducible analytical techniques [2-12]. Genomics projects have produced a large number of DNA sequences from a wide range of organisms, including humans and mammals. This "genomics revolution" has changed the concept of the comprehensive analysis of biological processes and systems. It is now hypothesized that biological processes and systems can be described based on the comparison of global, quantitative gene expression patterns from cells or tissues representing different states. The discovery of posttranscriptional mechanisms that control rate of synthesis and half-life of proteins and the ensuing nonpredictive correlation between mRNA and protein levels expressed by a particular gene indicate that direct measurement of protein expression also is essential for the analysis of biological processes and systems. Global analysis of gene expression at the protein level is now also termed proteomics. The standard method for quantitative proteome analysis combines protein separation by high resolution (isoelectric focusing/SDS-PAGE) two-dimensional gel electrophoresis (2DE) with mass spectrometric (MS) or tandem MS (MS/MS) identification of selected protein spots [5,9,11,13-16]. Important technical advances related to 2DE and protein MS have increased sensitivity, reproducibility, and throughput of proteome analysis while creating an integrated technology. Quantitation of protein expression in a proteome provides the first clue into how the cell responds to changes in its surrounding environments. The resulting over- or under-expressed proteins are deemed to play important roles in the precise regulation of cellular activities that are directly related to a given exogenous stimulus. Conventional 2DE, in combination with advanced mass spectrometric techniques, has facilitated the rapid characterization of thousands of proteins in a single polyacrylamide gel. The uniqueness of 2DE for easy visualisation of protein isoforms, using two physical parameters such as isoelectric point and molecular weight, renders this technology itself extremely informative. The method routinely analyzes more than 1000 different protein spots separated on a single two-dimensional gel and, thus, is well suited for the global analysis of protein expression in an organism. However, high-throughput quantitation of proteins from different cell lysates remains a challenging issue, owing to the poor reproducibility of 2DE, as well as low sensitivity and narrow linear dynamic ranges in the detection methods [17-21] Recent developments of fluorescent dyes, such as the different commercially available SYPRO dyes, partially addressed some of these problems [22-30]. These dyes detect as little as 1 ng of proteins, and at the same time they offer more than 1000-fold linear dynamic range. The more critical issue, however, is the reproducibility problem of 2DE. Even the identical protein samples that are run on two separate two-dimensional gels will normally produce very similar but not identical 2DE protein maps, owing to the gel-to-gel and operator-to-operator variations. This can be circumvented using multiplexing methods such as fluorescent two-dimensional "Difference Gel Electrophoresis" (2-D DIGE), which substantially reduces variability by displaying two or more complex protein mixtures labeled with different fluorescent dyes in a single 2D gel [21,31-38].

In this review, we focus on the latest developments in 2DE within the context of large-scale proteomics to reveal the advantages, limits and perspectives of the 2DE-based proteomic approach.

## A - Gel-based proteomic: from sample preparation to protein separation

### 1 - Protein solubilisation

In order to take advantage of the high resolution capacity of 2DE, proteins have to be completely denatured, disaggregated, reduced and solubilised to disrupt molecular interactions and to ensure that each spot represents an individual polypeptide.

Although a large number of standard protocols has been published, these protocols have to be adapted and further optimized for the type of sample (bacteria/yeast/mammalian cells; cells/tissue; animal/vegetal material; etc...) to be analyzed, as well as for the proteins of interest (cytosolic/nuclear; total "soluble" or membrane "insoluble" proteins; etc...).

After cell disruption, native proteins must be denatured and reduced to disrupt intra- and intermolecular interactions, and solubilized while maintaining the inherent charge properties. Sample solubilization is carried out using a buffer containing chaotropes (urea and/or thiourea), nonionic (Triton X-100) and/or zwitterionic detergents (CHAPS), reducing agents (DTT), carrier ampholytes and most of the time protease and phosphatase inhibitor cocktails are mandatory.

### 2 - The first dimension: isoelectric-focusing with immobilized pH gradients (IPGs)

Proteins are amphoteric molecules; they carry positive, negative or zero net charge, depending on their amino acid composition. The net charge of a protein is the sum of all the negative and positive charges. The isoelectric point (pI) of a protein is the specific pH at which the net charge of the protein is zero. Proteins are positively charged at pH values below their pI and are negatively charged at pH values above their pI. IEF is an electrophoretic separation based on this specific biochemical characteristic of proteins.

Basically, the first dimension of the 2DE is achieved with a "strip". It is a dry gel that is formed by the polymerization of acrylamide monomers, linked by bis-acrylamide with molecules of covalently linked immobilin. Immobilins are chemical components that are derived from acrylamide and have additional ionizable non-amphoteric functions. Immobilins of various pKa can create an immobilized pH gradient inside the acrylamide gel. Immobilin was developed by Professors Righetti and Görg at the beginning of the 1990s and is now widely used in 2DE because the IEF gradient is very stable over time and in a high electric field, and shows good reproducibility and a large capacity for separation [9,39-46].

The strip acrylamide gels are dried and cast on a plastic backing. Prior to use, they are rehydrated in a solution containing a pI-corresponding cocktail of carrier ampholytes and with the correct amount of proteins in the solubilization buffer. The carrier ampholytes are amphoteric molecules with a high buffering capacity near their pI. Commercial carrier ampholyte mixtures, which comprise species with pIs spanning a specific pH range, help the proteins to move.

When an electric field is applied, the negatively charged molecules (proteins and ampholytes) move towards the anode (positive/red electrode) and the positively charged molecules move towards the cathode (negative/black electrode). When the proteins are aligned according to their pI, the global net charge is zero and the protein is unable to move and is then focused. Focusing is achieved with a dedicated apparatus that is able to deliver up to 8000 or 10,000 V, but with a limitation in current intensity (50 μA maximum/strip) to reduce heat. The strips are usually first rehydrated without current for at least 5 h (passive rehydration), rehydrated with 50 V for 5 h (active rehydration) and then focused until at least 30 to 80 kV/h.

The equilibration step is critical for 2DE. In this step, the strips are saturated with sodium dodecyl sulfate (SDS), an anionic detergent that can denature proteins and form a negatively charged protein/SDS complex. The amount of SDS bound to a protein is directly proportional to the mass of the protein. Thus, proteins that are completely covered by negative charges are separated on the basis of molecular mass.

The equilibration solution also contains buffer, with urea and glycerol. Equilibration of the strips is achieved in two steps: (1) with an equilibration solution containing DTT, to maintain a reducing environment; and (2) with an equilibration solution containing iodoacetamide, to alkylate reduced thiol groups, preventing their re-oxidation during electrophoresis.

### 3 - The second dimension: SDS-PAGE

In SDS polyacrylamide gel electrophoresis (SDS-PAGE), migration is determined not by the intrinsic electric charge of polypeptides but by their molecular weight. The SDS-denatured and reduced proteins are separated according to an apparent molecular weight, in comparison with a molecular weight marker. A linear relationship between the logarithm of the molecular weight and the distance of migration of the proteins can be used; it depends essentially on the percentage of polyacrylamide.

Equilibrated strips are embedded with 1% (w/v) low-melting-point agarose in TRIS/Glycine/SDS running buffer and with 0.01% bromophenol blue on the top of the second dimension acrylamide gel. Gels are usually run with 1 or 2 W of current in the first hour, followed by 15 mA/gel overnight with a temperature regulation (10°C to 18°C). When the bromophenol blue migration front reaches the bottom of the gel, the second dimension is finished and the acrylamide gel can be removed from the glass plates.

### 4 - Gel staining

The gel must firstly be immersed in a fixation solution containing acid (phosphoric acid or acetic acid) and alcohol (ethanol or methanol) as a function of the staining protocol selected. Numerous stains can be used, but with very different costs [17]. Conventional "visible" dyes are Coomassie Blue, colloidal Coomassie Blue and silver nitrate, with quite different sensitivities: 50, 10 and 0.5 ng of detectable protein/spot respectively [17,20,25,47-51]. Commercially available fluorescent dyes, such as Sypro Ruby, Flamingo and Deep Purple, have sensitivities of about 1 ng of detectable protein/spot [21,23,26,28,52-55]. Fluorescent dyes have the advantage of a 4 log dynamic linear range but the disadvantage of being more expensive. In comparison with fluorescent dyes, silver nitrate stain has a dynamic linear range of only 1.5 log, and is not recommended for a gel comparison study.

### 5 - Bioinformatics analysis of 2-D images

**Figure 1:**
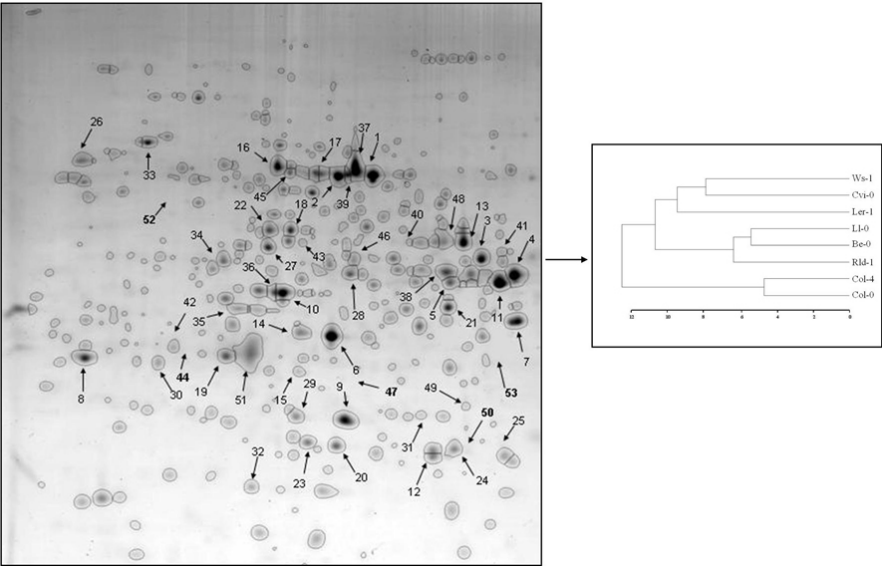
**Hierarchical clustering of *Arabidopsis thaliana *ecotypes based on 2DE spot amounts**. Soluble proteins from ecotype Col-0 were separated using pH 4-7 IPG in first dimension and 11% SDS-PAGE in second dimension. Proteins spots were visualized by colloidal Coomassie blue staining. The amount of each spot was estimated by its normalized volume as obtained by image analysis [62]. Euclidian distances were then computed for all spots to build the similarity matrix for ecotypes, and clustering was performed using the Ward's method to link the variables.

Stained gels are scanned on a "visible" or "fluorescent" scanner as a function of the staining protocol selected. The image can then be imported to specific software to be analysed and compared. For a comparison study, at least three repetitions of the same sample should be run; many migration artifacts can occur during 2DE and, to reduce such variability, a mean of several gels is essential. Software, such as Image Master, Progenesis, PDQuest and Samespots, can be used to detect spots and to compare the spot intensity between samples [53-60]. Spots of interest, *i.e*. spots specific to a sample or spots over-expressed on a condition/treatment, can be selected for further MS analysis. Several "computer-based" comparisons can be performed with a 2DE map. As a proteomic map is specific of a given cell, tissue or organism in a specific physiological condition, it is possible to compare not only one spot to one spot, but a set of spots to a set of spots, for example between two closed organisms. In a precedent study, we investigated the natural variation in the proteome among 8 *Arabidopsis thaliana *ecotypes, of which 3 were previously shown to display atypical responses to environmental stress [61]. The 2DE proteomic maps revealed important variations in terms of function between ecotypes [62]. Hierarchical clustering of proteomes according to either the amount of all anonymous spots, that of the 25 major spots or the functions of these major spots identified the same classes of ecotypes, and grouped the three atypical ecotypes (Fig. 1).

### 6 - Protein identification

**Figure 2:**
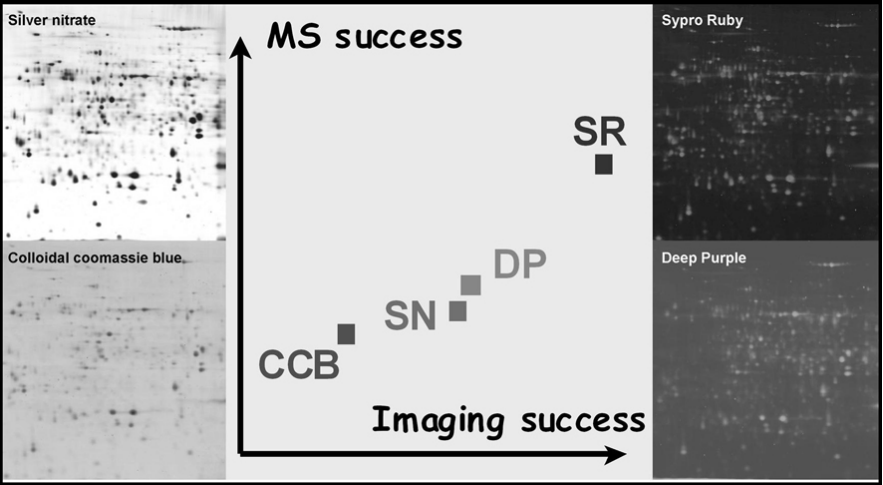
**Impact of staining procedures on mass spectrometry compatibility using visible stains and fluorescent dyes**. In these studies, the effects of protein spot properties were integrated to derive prediction of the MS results obtainable with the different dyes for all spots in the gels [24,49]. 100 μg of total protein extracts from *Arabidopsis thaliana *were focused on pI 4-7 range and separated on gels covering the 15-150 kDa range. By comparison to sensitivity properties of dyes, these simulations enable a first estimation of the overall proteomic capacity of dyes. They argue for a clear advantage in using fluorescent dyes, particularly SR, which cumulates high sensitivity, acceptable identification success on gels loaded with low protein amount, and constant protein sequence coverage. Abbreviations used: colloidal Coomassie blue (CCB), silver nitrate (SN), Sypro Ruby (SR), Deep Purple (DP).

To identify the proteins within the spots of interest (according to image analysis), a gel with a greater amount of protein is prepared. In this case, IEF step must be performed at least until 100 kV/h. The other steps of the 2DE are very similar to the previously described protocol. Colloidal Coomassie Blue or fluorescent dyes are recommended for the staining of the preparative gel, because they have good compatibility with MS [22,23,28,63]. In contrast, silver nitrate will give poor results, even if MS-compatible protocols are available [21,49,50]. It should be noted that a specific spot picker robot, able to work with fluorescence, is essential when working with fluorescent dyes. On a precedent study, we analyzed the total protein maps visualized when using classical visible stains and different fluorescent dyes [49]. For this purpose, a soluble extract from *Arabidopsis thaliana *was taken as a model of sequenced eukaryotic genome and resolved by 2-DE. Besides specificities in background quality, propensity to saturation, and staining reproducibility, large differences were observed between dyes in terms of sensitivity, especially for low abundance spots. The effects of the staining procedure on MALDI-TOF MS characterization were analyzed too on a set of 48 protein spots that were selected for their contrasting abundance, pI, and Mr. Gels were stained with either classical visible stains colloidal (Coomassie blue and silver nitrate), and different fluorescent dyes (Sypro Ruby and Deep Purple). It appeared that Sypro Ruby combined several favorable features: no dependence of the identification rate upon the physicochemical properties of proteins, no impact on frequency of missed cleavages, and a higher predicted identification rate (Fig. 2).

## B - Benefits of Gel-based proteomic

### 1 - A reduced gel to gel variation using "Difference in Gel Electrophoresis" (2D-DIGE)

Difference in gel electrophoresis (DIGE), first conceived by Unlu et al. in 1997, takes advantages of structurally similar cyanine-based dyes to label different pools of protein samples, which are then co-separated on a single 2DE gel [34].

The biggest advantage of DIGE over other two-dimensional- based technologies is that it enables the analysis of two or more protein samples simultaneously on a single 2DE [31,32,35,36]. Because the same proteins present in two different samples were prelabeled with two different dyes (i.e., Cy3 and Cy5, respectively), they could be combined and separated on the same 2DE without the loss of the relative protein abundance in the original samples. At the end of protein separation, the relative ratio of proteins in the two original samples could be readily obtained by comparing the fluorescence intensity of the same protein spots under different detection channels (e.g., Cy3 and Cy5) using a commercial fluorescence gel scanner. Because only one gel is used in DIGE, and the same proteins from two different protein samples comigrate as single spots, there is no need for the generation of "averaged" gels, as well as superimposition of different gels, making spot comparison and protein quantitation much more convenient and reliable. This makes DIGE potentially amendable for high-throughput proteomics applications.

DIGE has shown significant advantages over conventional 2DE in a number of applications. Up to three kinds of fluorescent cyanine dyes have been employed in DIGE, namely, Cy2, Cy3, and Cy5, which allows for simultaneous analysis of up to three different protein samples in a single gel. DIGE is a valuable method for high-throughput studies of protein expression profiles, providing opportunities to detect and quantify accurately "difficult" proteins, such as low-abundance proteins.

A problem in DIGE lies in the hydrophobicity of the cyanine dyes, which label the protein by reacting covalently, to a large extent, with surface- exposed lysines in the protein, and lead to removal of multiple charges from the protein. Consequently, this decreases the solubility of the labeled protein, and in some cases may lead to protein precipitation prior to gel electrophoresis. To address this problem, minimal labeling is generally employed in DIGE. Typically the labelling reaction is optimized such that only 1-5% of total lysines in a given protein are labeled. Alternatively, Shaw et al. have developed a new batch of DIGE Cy3 and Cy5 dyes, which label only free cysteines in a protein by "saturation" labeling [37]. This strategy offers greater sensitivity than the conventional DIGE method. The biggest drawback, however, is that it only labels proteins that contain free cysteines, meaning that a certain percentage of proteins in a proteome will not be labeled with this strategy, let alone downstream detection and characterization of these proteins.

### 2 - Characterization of protein isoforms

**Figure 3:**
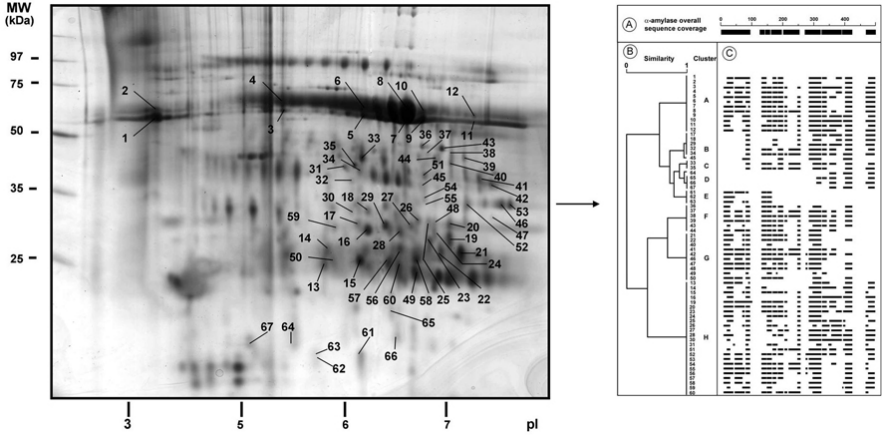
**Characterization of isoforms of alpha-amylase in human saliva**. A 2DE map of human salivary proteins was performed [68]. Proteins were resolved using pH 3-10NL IPG and 12% SDS-PAGE, and stained with colloidal Coomassie blue. Alpha-amylase spots subsequently identified by MALDI-TOF MS are indicated by respective spot number (left part). According to the mass features of alpha-amylase (right part) identified spots, (A) coverage of the alpha-amylase sequence by the total population of peptides identified (black boxes) in the different alpha-amylase spots, (B) simultaneous clustering of the 67 alpha-amylase spots according to the MW range measured on gels, the MS identification of peptides in the N-terminal and C-terminal and central regions of the sequence, (C) individual spot coverage of the alpha-amylase sequence by peptides identified (black boxes) by MALDI-TOF MS.

**Figure 4:**
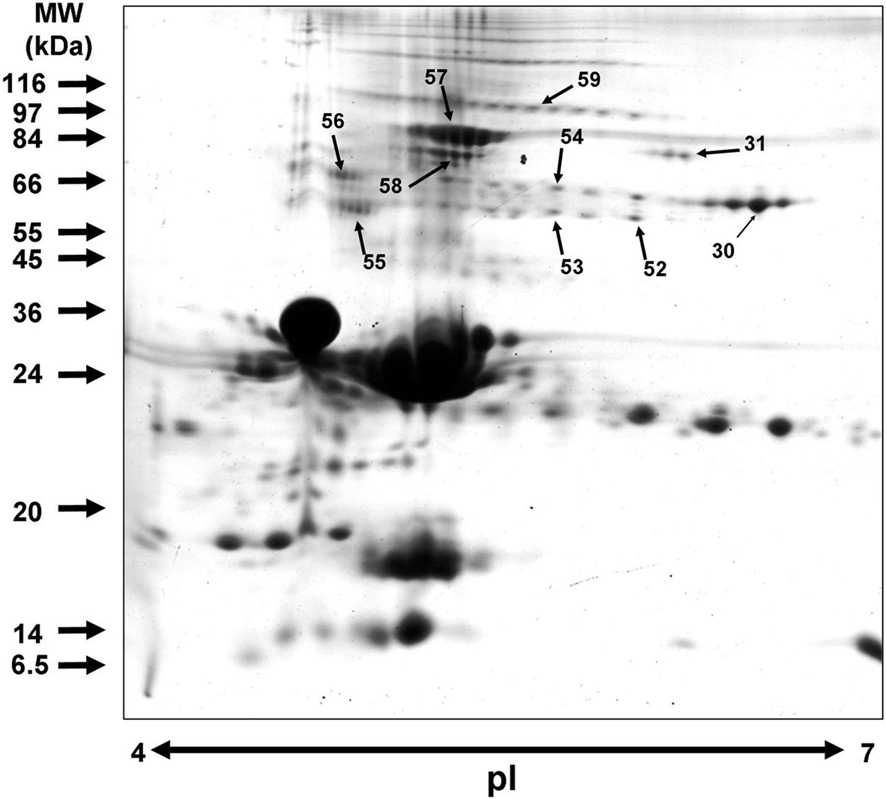
**Non-reducing 2DE for the study of disulfide-mediated interactions between proteins in raw milk**. 2DE of proteins in raw milk separated under non-reducing conditions using a 7-cm pH 4-7 pI range strip for the first dimension, and a 10 to 18% gradient acrylamide gel for the second dimension. The specific/interesting spots as indicated by arrows were submitted to MALDI-TOF mass to identify proteins involved in polymers [69].

An area of increasing interest in proteomics is the identification of post-translational modifications and/or spliced forms of a same gene or protein [64-67]. The process of determining whether a protein is expressed in a particular proteome has become a relatively simple task with the automation of the 'in-gel' digest and subsequent identification of the resulting peptides with mass spectrometers. Today, most proteins are identified by either assigning them definitive protein attributes, such as peptide masses generated by MALDI-TOF mass spectrometry and the short amino acid sequences generated by tandem MS. It is clear that when several spliced variants are present in a proteome, such approach for protein identification mostly characterizes peptides common to all spliced variants. In a precedent study, we used the advantages of 2DE separation to analyze alpha-amylase diversity in human saliva [68]. Because each alpha-amylase isoforms exist as a discrete purified protein, any information obtained from the analysis of this protein is unique to its original proteome (Fig. 3). 2DE was combined with systematic MALDI-TOF MS analysis and more than 140 protein spots identifying the alpha-amylase were shown to constitute a stable but very complex pattern. Careful analysis of mass spectra and simultaneous hierarchical clustering of the observed peptides and of the electrophoretic features of spots defined several groups of isoforms (Fig. 3 right part) with specific sequence characteristics, potentially related with special biological activities. In a recent study, 2DE separation was successfully used to analyze isoforms and polymers forms of bovine milk proteins [69]. A combination of reducing and non-reducing steps was used to reveal proteins polymers occurring before or after heat treatment of milk (Fig. 4). This original 2DE strategy revealed numerous disulfide-mediated interactions and was proposed to analyze reduction/oxidation of milk and dairy product proteins.

## C - Limits of "Gel-based proteomic"

### 1 - Membrane proteins

The resolution of membrane proteins remains an area of considerable concern in gel-based proteomics [70-75]. There remains an attitude that it is difficult or impossible to effectively resolve membrane proteins using 2DE. Indeed, few membrane proteins are seen on 2D gels when conventional sample-preparation methods are used. Membrane proteins are poorly soluble in the detergent/chaotrope conditions available for IEF, and are inherently insoluble in gel matrices under these conditions and thus are poorly resolved by IEF and subsequent 2DE. Fractionation, in combination with the correct solubilizing reagents, produces sample extracts that are highly enriched for membrane proteins. Sequential extraction of proteins from a sample by increasing protein solubility at each step is an effective strategy for first removing the more abundant soluble proteins and then for concentrating the less abundant and less soluble membrane proteins. A specific 2DE strategy, using the cationic detergent benzyldimethyl-n-hexadecylammonium chloride in the first and the anionic detergent SDS in the second dimension, was successfully used to analyze membranes proteins in various systems [76-78].

### 2 - Low-abundance proteins

Low-abundance proteins are rarely seen on traditional 2D maps because large quantities of abundant soluble proteins obscure their detection [21,79-81]. Most 2DE-based proteomic studies employ a 'one-extract-one-gel' approach and the majority of proteins identified in these studies are in high abundance. These low-abundance proteins are considered to be some of the most important, including regulatory proteins, signal transduction proteins and receptors. Consequently, the analysis of low-abundance proteins is becoming increasingly common in cellular proteomics. The dynamic range of protein concentration can differ considerably between biological samples. For yeast, the most abundant proteins are present at around 2 000 000 copies per cell, whereas the least abundant proteins are present at around 100 copies per cell, a dynamic range of only 4 orders of magnitude. However, in plasma, the predicted dynamic range of proteins is up to 12 orders of magnitude. Analysis of individual compartments not only provides information on protein localization, but also allows detection of protein populations otherwise not detectable in whole cell proteomes. Detection of the low-abundance proteins requires most of the time removal of abundant proteins from the sample. For example, the complexity of the serum and plasma proteome presents extreme analytical challenges in comprehensive analysis due to the wide dynamic range of protein concentrations. Therefore, robust sample preparation methods remain one of the important steps in the proteome characterization workflow. A specific depletion of high-abundant proteins from human serum and plasma using affinity columns is of particular interest to improve dynamic range for proteomic analysis and enable the identification of low-abundant plasma proteins [80,82]. On another hand, IPG technology can be used with narrow (2-3 pH units) and very narrow (~1 pH unit) gradients that enable many more proteins to be resolved. Indeed, the advent of immobilized pH gradients has greatly improved the reproducibility of 2D gels and has made it easier for new users to implement this technology. The loading capacity of narrow-range IPGs is considerably higher than broad-range IPGs, thus enabling the visualization and identification of previously unseen proteins. Sub-fractionation can be used to improve the recovery of low-abundance proteins too. For example, membrane preparation methods are commercially available and allow a specific separation between abundant/soluble proteins and membrane/low-abundance proteins. More recently, a system is available to perform a specific depletion of high-abundant proteins and a reduction of protein concentration differences [82,83]. The protein population is "equalized", by sharply reducing the concentration of the most abundant components, while simultaneously enhancing the concentration of the most dilute species.

### 3 - Alkaline proteins

Alkaline proteins were particularly difficult to resolve on 2D gels. First, the most common commercially available pH gradients, until recently, were pH 4-7 and pH 3-10 and these do not provide significant alkaline-protein resolution. As more alkaline pH range immobilized pH gradients become commercially available, resolution of proteins in IPGs up to pH 12 has been demonstrated. Strongly alkaline proteins such as ribosomal and nuclear proteins with closely related pIs between 10.5 and 11.8 were focused to the steady state by using 3-12, 6-12 and 9- 12 pI ranges [84-86]. For highly resolved 2-D patterns, different optimization steps with respect to pH engineering and gel composition were necessary, such as the substitution of dimethylacrylamide for acrylamide, the addition near the cathode of a paper strip soaked with DTT providing a continuous influx of DTT to compensate for the loss of DTT [41,45], and the addition of isopropanol to the IPG rehydration solution in order to suppress the reverse electroendosmotic flow which causes highly streaky 2-D patterns in narrow pH range IPGs 9-12 and 10-12 [86].

### 4 - High molecular weight proteins

Conventional 2DE, using SDS-PAGE as second dimension is not very useful to visualize high-molecular-weight (HMW) proteins. Even with gradient acrylamide gels, it is very difficult to obtain a good separation of proteins up to 250 kDa. HMW proteins and protein-complexes can be analysed using agarose gel IEF [87]. Agarose 2-DE is sufficiently good at separating HMM proteins with molecular masses as large as 500 kDa from various diseased tissues and cells [88]. Indeed, this method was successfully used to analyze HMW complexes from yeast [87], HMW proteins from human plasma [89], hepatocellular carcinoma, prostate and colorectal cancer [88,90-92].

## Conclusion

Thanks to its high resolving power and its large sample loading capacity, 2DE allows several hundred proteins to be displayed simultaneously on a single gel, producing a direct and global view of a sample proteome at a given time point. Reference maps of numerous distinct samples have now been published, providing, to researchers worldwide, standardized libraries of proteins known to be present in these samples. But 2DE has some limitations that must be taken into account. Despite maximal precautions, there will be some degree of gel-to-gel and run-to-run variability in the expression of the same protein set, which could be overcome by maintaining a variability coefficient of reference spots as low as possible. It can be largely circumvented using a DIGE strategy. Additionally, some proteins may escape the capabilities of conventional 2DE for several reasons, including the poor solubility of membrane proteins and out of range characteristics of extreme proteins such as high or low pI and molecular weight. Despite all these drawbacks, 2DE can demonstrate changes in relative abundance of visualized proteins and can detect protein isoforms, variants, polymer complexes and posttranslational modifications. Quantitative proteomics can be achieved by assessing differences in protein expression across gels using 2DE dedicated software and proteins in varying spots can be identified by MS. The uniqueness of 2DE for easy visualization of protein isoforms renders this technology itself extremely informative and it is currently the most rapid method for direct targeting of protein expression differences.

## Competing interests

The author declares that they have no competing interests.

## Authors' contributions

FC carried out all of this work. All authors read and approved the final manuscript.

## References

[B1] O'Farrell PH High resolution two-dimensional electrophoresis of proteins Journal of Biological Chemistry 1975 250 4007 4021 236308 PMC2874754

[B2] Wan JH He FC Technical development of proteomics Chinese Science Bulletin 1999 44 1441 1447 10.1007/BF03183559

[B3] Gromov PS Celis JE From genomics to proteomics Molecular Biology 2000 34 508 520 10.1007/BF02759560 11042852

[B4] Govorun VM Archakov AI Proteomic technologies in modern biomedical science Biochemistry-Moscow 2002 67 1109 1123 10.1023/A:1020959106412 12460109

[B5] Garfin DE Two-dimensional gel electrophoresis: an overview Trac-Trends in Analytical Chemistry 2003 22 263 272 10.1016/S0165-9936(03)00506-5

[B6] Collinsova M Jiracek J Current development in proteomics Chemicke Listy 2004 98 1112 1118

[B7] Bradshaw RA Burlingame AL From proteins to proteomics Iubmb Life 2005 57 267 272 10.1080/15216540500091536 16036609

[B8] Bergh G Van den Arckens L Recent advances in 2D electrophoresis: an array of possibilities Expert Review of Proteomics 2005 2 243 252 10.1586/14789450.2.2.243 15892568

[B9] Carrette O Burkhard PR Sanchez JC Hochstrasser DF State-of-the-art two-dimensional gel electrophoresis: a key tool of proteomics research Nature Protocols 2006 1 812 823 10.1038/nprot.2006.104 17406312

[B10] Bergeron JJM Bradshaw RA What has proteomics accomplished? Molecular & Cellular Proteomics 2007 6 1824 1826 17913850

[B11] Penque D Two-dimensional gel electrophoresis and mass spectrometry for biomarker discovery Proteomics Clinical Applications 2009 3 155 172 10.1002/prca.200800025 26238616

[B12] Bergh G Van den Arckens L High Resolution Protein Display by Two-Dimensional Electrophoresis Current Analytical Chemistry 2009 5 106 115 10.2174/157341109787846199

[B13] HumpherySmith I Cordwell SJ Blackstock WP Proteome research: Complementarity and limitations with respect to the RNA and DNA worlds Electrophoresis 1997 18 1217 1242 10.1002/elps.1150180804 9298643

[B14] Celis JE Gromov P 2D protein electrophoresis: can it be perfected? Current Opinion in Biotechnology 1999 10 16 21 10.1016/S0958-1669(99)80004-4 10047502

[B15] Ong SE Pandey A An evaluation of the use of two-dimensional gel electrophoresis in proteomics Biomolecular Engineering 2001 18 195 205 10.1016/S1389-0344(01)00095-8 11911086

[B16] Lopez JL Two-dimensional electrophoresis in proteome expression analysis Journal of Chromatography B-Analytical Technologies in the Biomedical and Life Sciences 2007 849 190 202 10.1016/j.jchromb.2006.11.049 17188947

[B17] Chevalier F Rofidal V Rossignol M Visible and fluorescent staining of two-dimensional gels Methods in Molecular Biology 2007 355 145 156 17093309 10.1385/1-59745-227-0:145

[B18] Harris LR Churchward MA Butt RH Coorssen JR Assessing detection methods for gel-based proteomic analyses Journal of Proteome Research 2007 6 1418 1425 10.1021/pr0700246 17367184

[B19] Volkova KD Kovalska VB Yarmoluk SM Modern techniques for protein detection on polyacrylamide gels: problems arising from the use of dyes of undisclosed structures for scientific purposes Biotechnic & Histochemistry 2007 82 201 208 10.1080/10520290701707660 18074266

[B20] Smejkal GB The Coomassie chronicles: past, present and future perspectives in polyacrylamide gel staining Expert Review of Proteomics 2004 1 381 387 10.1586/14789450.1.4.381 15966833

[B21] Patton WF Detection technologies in proteome analysis Journal of Chromatography B-Analytical Technologies in the Biomedical and Life Sciences 2002 771 3 31 10.1016/S1570-0232(02)00043-0 12015990

[B22] Cong WT Hwang SY Jin LT Choi JK Sensitive fluorescent staining for proteomic analysis of proteins in 1-D and 2-D SDS-PAGE and its comparison with SYPRO Ruby by PMF Electrophoresis 2008 29 4304 4315 10.1002/elps.200800150 19016505

[B23] Ball MS Karuso P Mass spectral compatibility of four proteomics stains Journal of Proteome Research 2007 6 4313 4320 10.1021/pr070398z 17929854

[B24] Chevalier F Centeno D Rofidal V Tauzin M Martin O Sommerer N Rossignol M Different impact of staining procedures using visible stains and fluorescent dyes for large-scale investigation of proteomes by MALDI-TOF mass spectrometry Journal of Proteome Research 2006 5 512 520 10.1021/pr050194n 16512665

[B25] Lanne B Panfilov O Protein staining influences the quality of mass spectra obtained by peptide mass fingerprinting after separation on 2-D gels. A comparison of staining with coomassie brilliant blue and SYPRO Ruby Journal of Proteome Research 2005 4 175 179 10.1021/pr040005l 15707373

[B26] Lamanda A Zahn A Roder D Langen H Improved Ruthenium II tris (bathophenantroline disulfonate) staining and destaining protocol for a better signal-to-background ratio and improved baseline resolution Proteomics 2004 4 599 608 10.1002/pmic.200300587 14997483

[B27] White IR Pickford R Wood J Skehel JM Gangadharan B Cutler P A statistical comparison of silver and SYPRO Ruby staining for proteomic analysis Electrophoresis 2004 25 3048 3054 10.1002/elps.200405947 15349947

[B28] Berggren KN Schulenberg B Lopez MF Steinberg TH Bogdanova A Smejkal G Wang A Patton WF An improved formulation of SYPRO Ruby protein gel stain: Comparison with the original formulation and with a ruthenium II tris (bathophenanthroline disulfonate) formulation Proteomics 2002 2 486 498 10.1002/1615-9861(200205)2:5<486::AID-PROT486>3.0.CO;2-X 11987123

[B29] Rabilloud T Strub JM Luche S van Dorsselaer A Lunardi J Comparison between Sypro Ruby and ruthenium II tris (bathophenanthroline disulfonate) as fluorescent stains for protein detection in gels Proteomics 2001 1 699 704 10.1002/1615-9861(200104)1:5<699::AID-PROT699>3.0.CO;2-C 11678039

[B30] Berggren K Steinberg TH Lauber WM Carroll JA Lopez MF Chernokalskaya E Zieske L Diwu ZJ Haugland RP Patton WF A luminescent ruthenium complex for ultrasensitive detection of proteins immobilized on membrane supports Analytical Biochemistry 1999 276 129 143 10.1006/abio.1999.4364 10603235

[B31] Karp NA Feret R Rubtsov DV Lilley KS Comparison of DIGE and post-stained gel electrophoresis with both traditional and SameSpots analysis for quantitative proteomics Proteomics 2008 8 948 960 10.1002/pmic.200700812 18246571

[B32] Hrebicek T Duerrschmid K Auer N Bayer K Rizzi A Effect of CyDye minimum labeling in differential gel electrophoresis on the reliability of protein identification Electrophoresis 2007 28 1161 1169 10.1002/elps.200600639 17340647

[B33] Karp NA McCormick PS Russell MR Lilley KS Experimental and statistical considerations to avoid false conclusions in proteomics studies using differential in-gel electrophoresis Molecular & Cellular Proteomics 2007 6 1354 1364 10.1074/mcp.M600274-MCP200 17513293

[B34] Viswanathan S Unlu M Minden JS Two-dimensional difference gel electrophoresis Nature Protocols 2006 1 1351 1358 10.1038/nprot.2006.234 17406422

[B35] Wheelock AM Morin D Bartosiewicz M Buckpitt AR Use of a fluorescent internal protein standard to achieve quantitative two-dimensional gel electrophoresis Proteomics 2006 6 1385 1398 10.1002/pmic.200402083 16429456

[B36] Gade D Thiermann J Markowsky D Rabus R Evaluation of two-dimensional difference gel electrophoresis for protein profiling Journal of Molecular Microbiology and Biotechnology 2003 5 240 251 10.1159/000071076 12867748

[B37] Shaw J Rowlinson R Nickson J Stone T Sweet A Williams K Tonge R Evaluation of saturation labelling two-dimensional difference gel electrophoresis fluorescent dyes Proteomics 2003 3 1181 1195 10.1002/pmic.200300439 12872219

[B38] Westermeier R Loyland S Asbury R Proteomics technology Journal of Clinical Ligand Assay 2002 25 242 252

[B39] Bjellqvist B Ek K Righetti PG Gianazza E Gorg A Westermeier R Postel W Isoelectric-focusing in immobilized ph gradients - principle, methodology and some applications Journal of Biochemical and Biophysical Methods 1982 6 317 339 10.1016/0165-022X(82)90013-6 7142660

[B40] Righetti PG Castagna A Hamdan M Recent trends in proteome analysis Advances in Chromatography 2003 42 269 321 *Advances in Chromatography* 12611023

[B41] Altland K Becher P Rossmann U Bjellqvist B Isoelectric-focusing of basic-proteins - the problem of oxidation of cysteines Electrophoresis 1988 9 474 485 10.1002/elps.1150090906 3243245

[B42] Bouchal P Kucera I Two-dimensional electrophoresis in proteomics: Principles and applications Chemicke Listy 2002 97 29 36

[B43] Friedman DB Hoving S Westermeier R Isoelectric focusing and two-dimensional gel electrophoresis Guide to Protein Purification 2009 466 Second 515 540 *Methods in Enzymology* 10.1016/S0076-6879(09)63030-5 19892190

[B44] Gorg A Postel W Gunther S The current state of two-dimensional electrophoresis with immobilized ph gradients Electrophoresis 1988 9 531 546 10.1002/elps.1150090913 3072185

[B45] Gorg A Boguth G Obermaier C Posch A Weiss W 2-dimensional polyacrylamide-gel electrophoresis with immobilized ph gradients in the first dimension (IPG-DALT) - the state-of-the-art and the controversy of vertical versus horizontal systems Electrophoresis 1995 16 1079 1086 10.1002/elps.11501601183 7498150

[B46] Gorg A Weiss W Dunn MJ Current two-dimensional electrophoresis technology for proteomics Proteomics 2004 4 3665 3685 10.1002/pmic.200401031 15543535

[B47] Rabilloud T A comparison between low background silver diammine and silver-nitrate protein stains Electrophoresis 1992 13 429 439 10.1002/elps.1150130190 1425556

[B48] Neuhoff V Stamm R Pardowitz I Arold N Ehrhardt W Taube D Essential problems in quantification of proteins following colloidal staining with coomassie brilliant blue dyes in polyacrylamide gels, and their solution Electrophoresis 1990 11 101 117 10.1002/elps.1150110202 1692528

[B49] Chevalier F Rofidal V Vanova P Bergoin A Rossignol M Proteomic capacity of recent fluorescent dyes for protein staining Phytochemistry 2004 65 1499 1506 10.1016/j.phytochem.2004.04.019 15276447

[B50] Mortz E Krogh TN Vorum H Gorg A Improved silver staining protocols for high sensitivity protein identification using matrix-assisted laser desorption/ionization-time of flight analysis Proteomics 2001 1 1359 1363 10.1002/1615-9861(200111)1:11<1359::AID-PROT1359>3.0.CO;2-Q 11922595

[B51] Jin LT Li XK Cong WT Hwang SY Choi JK Previsible silver staining of protein in electrophoresis gels with mass spectrometry compatibility Analytical Biochemistry 2008 383 137 143 10.1016/j.ab.2008.04.048 18804088

[B52] Nebrich G Liegmann H Wacker M Herrmann M Sagi D Landowsky A Klose J Proteomer, a novel software application for management of proteomic 2DE-gel data-II application Molecular & Cellular Proteomics 2005 4 S296 S296

[B53] Wheelock AM Buckpitt AR Software-induced variance in two-dimensional gel electrophoresis image analysis Electrophoresis 2005 26 4508 4520 10.1002/elps.200500253 16315176

[B54] Maurer MH Software analysis of two-dimensional electrophoretic gels in proteomic experiments Current Bioinformatics 2006 1 255 262 10.2174/157489306777011969

[B55] Wheelock AM Goto S Effects of post-electrophoretic analysis on variance in gel-based proteomics Expert Review of Proteomics 2006 3 129 142 10.1586/14789450.3.1.129 16445357

[B56] Rosengren AT Salmi JM Aittokallio T Westerholm J Lahesmaa R Nyman TA Nevalainen OS Comparison of PDQuest and Progenesis software packages in the analysis of two-dimensional electrophoresis gels Proteomics 2003 3 1936 1946 10.1002/pmic.200300544 14625856

[B57] Nebrich G Liegmann H Wacker M Herrmann M Sagi D Landowsky A Klose J Proteomer, a novel software application for management of proteomic 2DE-gel data-II application Molecular & Cellular Proteomics 2005 4 S296 S296

[B58] Clark BN Gutstein HB The myth of automated, high-throughput two-dimensional gel analysis Proteomics 2008 8 1197 1203 10.1002/pmic.200700709 18283661

[B59] Panchaud A Affolter M Moreillon P Kussmann M Experimental and computational approaches to quantitative proteomics: Status quo and outlook Journal of Proteomics 2008 71 19 33 10.1016/j.jprot.2007.12.001 18541471

[B60] Kang YY Techanukul T Mantalaris A Nagy JM Comparison of Three Commercially Available DIGE Analysis Software Packages: Minimal User Intervention in Gel-Based Proteomics Journal of Proteome Research 2009 8 1077 1084 10.1021/pr800588f 19133722

[B61] Chevalier F Pata M Nacry P Doumas P Rossignol M Effects of phosphate availability on the root system architecture: large-scale analysis of the natural variation between Arabidopsis accessions Plant Cell and Environment 2003 26 1839 1850 10.1046/j.1365-3040.2003.01100.x

[B62] Chevalier F Martin O Rofidal V Devauchelle AD Barteau S Sommerer N Rossignol M Proteomic investigation of natural variation between Arabidopsis ecotypes Proteomics 2004 4 1372 1381 10.1002/pmic.200300750 15188405

[B63] Nock CM Ball MS White IR Skehel JM Bill L Karuso P Mass spectrometric compatibility of Deep Purple and SYPRO Ruby total protein stains for high-throughput proteomics using large-format two-dimensional gel electrophoresis Rapid Communications in Mass Spectrometry 2008 22 881 886 10.1002/rcm.3483 18293286

[B64] Holland JW Deeth HC Alewood PF Proteomic analysis of K-casein micro-heterogeneity Proteomics 2004 4 743 752 10.1002/pmic.200300613 14997496

[B65] Schulenberg B Goodman TN Aggeler R Capaldi RA Patton WF Characterization of dynamic and steady-state protein phosphorylation using a fluorescent phosphoprotein gel stain and mass spectrometry Electrophoresis 2004 25 2526 2532 10.1002/elps.200406007 15300772

[B66] Ahrer K Jungbauer A Chromatographic and electrophoretic characterization of protein variants Journal of Chromatography B-Analytical Technologies in the Biomedical and Life Sciences 2006 841 110 122 10.1016/j.jchromb.2006.05.044 16872917

[B67] Poth AG Deeth HC Alewood PF Holland JW Analysis of the Human Casein Phosphoproteome by 2-D Electrophoresis and MALDI-TOF/TOF MS Reveals New Phosphoforms Journal of Proteome Research 2008 7 5017 5027 10.1021/pr800387s 18847231

[B68] Hirtz C Chevalier F Centeno D Rofidal V Egea JC Rossignol M Sommerer N de Periee DD MS characterization of multiple forms of alpha-amylase in human saliva Proteomics 2005 5 4597 4607 10.1002/pmic.200401316 16294315

[B69] Chevalier F Hirtz C Sommerer N Kelly AL Use of Reducing/Nonreducing Two-Dimensional Electrophoresis for the Study of Disulfide-Mediated Interactions between Proteins in Raw and Heated Bovine Milk Journal of Agricultural and Food Chemistry 2009 57 5948 5955 10.1021/jf900518n 19526987

[B70] Luche S Santoni V Rabilloud T Evaluation of nonionic and zwitterionic detergents as membrane protein solubilizers in two-dimensional electrophoresis Proteomics 2003 3 249 253 10.1002/pmic.200390037 12627377

[B71] Santoni V Kieffer S Desclaux D Masson F Rabilloud T Membrane proteomics: Use of additive main effects with multiplicative interaction model to classify plasma membrane proteins according to their solubility and electrophoretic properties Electrophoresis 2000 21 3329 3344 10.1002/1522-2683(20001001)21:16<3329::AID-ELPS3329>3.0.CO;2-F 11079553

[B72] Santoni V Molloy M Rabilloud T Membrane proteins and proteomics: Un amour impossible? Electrophoresis 2000 21 1054 1070 10.1002/(SICI)1522-2683(20000401)21:6<1054::AID-ELPS1054>3.0.CO;2-8 10786880

[B73] Santoni V Doumas P Rouquie D Mansion M Rabilloud T Rossignol M Large scale characterization of plant plasma membrane proteins Biochimie 1999 81 655 661 10.1016/S0300-9084(99)80122-9 10433119

[B74] Santoni V Rabilloud T Doumas P Rouquie D Mansion M Kieffer S Garin J Rossignol M Towards the recovery of hydrophobic proteins on two-dimensional electrophoresis gels Electrophoresis 1999 20 705 711 10.1002/(SICI)1522-2683(19990101)20:4/5<705::AID-ELPS705>3.0.CO;2-Q 10344236

[B75] Chevallet M Santoni V Poinas A Rouquie D Fuchs A Kieffer S Rossignol M Lunardi J Garin J Rabilloud T New zwitterionic detergents improve the analysis of membrane proteins by two-dimensional electrophoresis Electrophoresis 1998 19 1901 1909 10.1002/elps.1150191108 9740050

[B76] Moebius J Zahedi RP Lewandrowski U Berger C Walter U Sickmann A The human platelet membrane proteome reveals several new potential membrane proteins Molecular & Cellular Proteomics 2005 4 1754 1761 10.1074/mcp.M500209-MCP200 16081409

[B77] Zahedi RP Meisinger C Sickmann A Two-dimensional benzyldimethyl-n-hexadecylammonium chloride/SDS-PAGE for membrane proteomics Proteomics 2005 5 3581 3588 10.1002/pmic.200401214 16075424

[B78] Dreger M Bengtsson L Schoneberg T Otto H Hucho F Nuclear envelope proteomics: Novel integral membrane proteins of the inner nuclear membrane Proceedings of the National Academy of Sciences of the United States of America 2001 98 11943 11948 10.1073/pnas.211201898 11593002 PMC59747

[B79] Yamada M Murakami K Wallingford JC Yuki Y Identification of low-abundance proteins of bovine colostral and mature milk using two-dimensional electrophoresis followed by microsequencing and mass spectrometry Electrophoresis 2002 23 1153 1160 10.1002/1522-2683(200204)23:7/8<1153::AID-ELPS1153>3.0.CO;2-Y 11981865

[B80] Greenough C Jenkins RE Kitteringham NR Pirmohamed M Park BK Pennington SR A method for the rapid depletion of albumin and immunoglobulin from human plasma Proteomics 2004 4 3107 3111 10.1002/pmic.200300815 15378708

[B81] Ahmed N Rice GE Strategies for revealing lower abundance proteins in two-dimensional protein maps Journal of Chromatography B-Analytical Technologies in the Biomedical and Life Sciences 2005 815 39 50 10.1016/j.jchromb.2004.10.070 15652797

[B82] Righetti PG Castagna A Boschetti E Lomas L Equalizer beads; The quest for a democratic proteome Molecular & Cellular Proteomics 2005 4 S12 S12 10.1002/pmic.200500904 16800034

[B83] Righetti PG Boschetti E Lomas L Citterio A Protein Equalizer (TM) Technology: The quest for a democratic proteome Proteomics 2006 6 3980 3992 10.1002/pmic.200500904 16800034

[B84] Drews O Reil G Parlar H Gorg A Setting up standards and a reference map for the alkaline proteome of the Gram-positive bacterium *Lactococcus lactis* Proteomics 2004 4 1293 1304 10.1002/pmic.200300720 15188396

[B85] Wildgruber R Reil G Drews O Parlar H Gorg A Web-based two-dimensional database of *Saccharomyces cerevisiae *proteins using immobilized pH gradients from pH 6 to pH 12 and matrix-assisted laser desorption/ionization-time of flight mass spectrometry Proteomics 2002 2 727 732 10.1002/1615-9861(200206)2:6<727::AID-PROT727>3.0.CO;2-2 12112855

[B86] Gorg A Obermaier C Boguth G Csordas A Diaz JJ Madjar JJ Very alkaline immobilized pH gradients for two-dimensional electrophoresis of ribosomal and nuclear proteins Electrophoresis 1997 18 328 337 10.1002/elps.1150180306 9150910

[B87] Yokoyama R Iwafune Y Kawasaki H Hirano H Isoelectric focusing of high-molecular-weight protein complex under native conditions using agarose gel Analytical Biochemistry 2009 387 60 63 10.1016/j.ab.2009.01.009 19454258

[B88] Oh-Ishi M Maeda T Disease proteomics of high-molecular-mass proteins by two-dimensional gel electrophoresis with agarose gels in the first dimension (Agarose 2-DE) Journal of Chromatography B-Analytical Technologies in the Biomedical and Life Sciences 2007 849 211 222 10.1016/j.jchromb.2006.10.064 17141588

[B89] Jin Y Manabe T Performance of agarose IEF gels as the first dimension support for non-denaturing micro-2-DE in the separation of high-molecular-mass plasma proteins and protein complexes Electrophoresis 2009 30 939 948 10.1002/elps.200800539 19309012

[B90] Seimiya M Tomonaga T Matsushita K Sunaga M Oh-Ishi M Kodera Y Maeda T Takano S Togawa A Yoshitomi H Identification of novel immunohistochemical tumor markers for primary hepatocellular carcinoma; Clathrin heavy chain and formiminotransferase cyclodeaminase Hepatology 2008 48 519 530 10.1002/hep.22364 18571811

[B91] Kuruma H Egawa S Oh-Ishi M Kodera Y Satoh M Chen WG Okusa H Matsumoto K Maeda T Baba S High molecular mass proteome of androgen-independent prostate cancer Proteomics 2005 5 1097 1112 10.1002/pmic.200401115 15712236

[B92] Tomonaga T Matsushita K Yamaguchi S Oh-Ishi M Kodera Y Maeda T Shimada H Ochiai T Nomura F Identification of altered protein expression and post-translational modifications in primary colorectal cancer by using agarose two-dimensional gel electrophoresis Clinical Cancer Research 2004 10 2007 2014 10.1158/1078-0432.CCR-03-0321 15041719

